# Supplementary material for: Healthy Urban Environmental Features for Poverty Resilience: The Case of Detroit, USA
Source: Int J Environ Res Public Health. 2021 Jun 29;18(13):6982. doi: 10.3390/ijerph18136982 (PMC8296987; doi:10.3390/ijerph18136982)
Supplement: Supplementary file 1 [file ijerph-18-06982-s001.zip › ijerph-1249308-supplementary.pdf]

Supplementary Material

for

Healthly Urban Environmental Features for Poverty Resilience: The Case of Detroit, USA.

Patricia Leandro-Reguillo, Amy L. Stuart

Index

Table S1. Data sources, specifications, and time frame

Table S2. Spearman correlation of select explanatory variables with all other covariates at the cell scale.

Table S3. Spearman correlation of select explanatory variables with all other covariates at the buffer scale.

Figure S1. The study area of metropolitan Detroit, with census tract outlines overlaid by the 1x1 mile standardized grid of cells used for correlation analyses.

Figure S2. Histogram and normal quantile plot of the difference in median household income (DMHI) between 2017 and 2013.

Figure S3. Scatter plots of DMHI and several urban environmental features.

Table S1. Data sources, specifications, and time frame.

	Environmental and urban factors	Description	Year of data*	Source	Webpage
1	Train/bus stations	Train and bus main stations (points)	2017	Google Earth	https://www.google.com/intl/es-419/earth/
2	Smartbus lines	Smartbus lines (# lines)	1970 (2016)	City of Detroit/ Detroit Open Data	https://data.detroitmi.gov
3	Bike lanes	Finished bike lanes (# lines)†	2014 (2015)	Data Driven Detroit	http://portal.datadrivendetroit.org/
4	Bus stops	Bus stops (points)	2016	City of Detroit/ Detroit Open Data	https://data.detroitmi.gov
5	Airports	Airport locations (points)	2017	SEMCOG	http://maps-semcog.opendata.arcgis.com
6	Toxic releases	Toxic releases inventory sites (points)	2015	EPA Toxic Release Inventory	https://www.epa.gov/toxics-release-inventory-tri-program
7	Railways	Railways (lines)	2017	SEMCOG	http://maps-semcog.opendata.arcgis.com
8	AADT 5000	Roads with annual average daily traffic exceeding 5000 vehicle per day (lines)	2017	SEMCOG	http://maps-semcog.opendata.arcgis.com
9	Industry	Industrial land uses (polygons)	2015	SEMCOG	http://maps-semcog.opendata.arcgis.com
10	Brownfields	Properties with the potential presence of hazardous substances (points)	2015	Data Driven Detroit	http://portal.datadrivendetroit.org/
11	NAASO2	USEPA Nonattainment areas for SO_2 (polygons)	2010	EPA Air Data	https://www.epa.gov/outdoor-air-quality-data/interactive-map-air-quality-monitors
12	Poor pavement	Roads with poor pavement (lines)	2017	SEMCOG	http://maps-semcog.opendata.arcgis.com
13	Demolitions	Properties affected by demolitions (points)	2009 (2015)	Data Driven Detroit	http://portal.datadrivendetroit.org/
14	Vacancies	Buildings that are very likely vacant (points)	2014 (2015)	Data Driven Detroit	http://portal.datadrivendetroit.org/
15	Public libraries	Public libraries (points)	2015	Data Driven Detroit	http://portal.datadrivendetroit.org/
16	Schools	Open schools (points)	2014 (2015)	Data Driven Detroit	http://portal.datadrivendetroit.org/
17	Colleges	Colleges and universities (points)	2014 (2015)	Data Driven Detroit	http://portal.datadrivendetroit.org/
18	Head Start	Head Start children's center locations (points)	2016	City of Detroit/ Detroit Open Data	https://data.detroitmi.gov
19	Police stations	Open police stations (points)	2012 (2015)	Data Driven Detroit	http://portal.datadrivendetroit.org/
20	Fire stations	Open fire stations (points)	2016	Data Driven Detroit	http://portal.datadrivendetroit.org/
21	Commercial	Office and commercial land uses (polygons)	2015	SEMCOG	http://maps-semcog.opendata.arcgis.com
22	Recreation centers	Open recreation centers (polygons)	2012 (2015)	Data Driven Detroit	http://portal.datadrivendetroit.org/
23	Hospitals	Hospitals (points)	2015 (2017)	Data Driven Detroit	http://portal.datadrivendetroit.org/
24	Health centers	Open health centers (points)	2017	City of Detroit/ Detroit Open Data	https://data.detroitmi.gov
25	Groceries	Open grocery stores (points)	2013 (2015)	Data Driven Detroit	http://portal.datadrivendetroit.org/
26	Parks	Public Parks (points)	2010 (2015)	Data Driven Detroit	http://portal.datadrivendetroit.org/
27	Cemeteries	Cemeteries (points)	2010 (2017)	Data Driven Detroit	http://portal.datadrivendetroit.org/

*Date of last nominal data update is in parenthesis, but values have not changed from the first-listed year. †Includes lanes, greenways, and sharrows.

Table S2. Spearman correlation of select explanatory variables with all other covariates at the cell scale.

covariate	bike lanes*	railways	industry*	brownfields*	poor pavement	libraries	schools*	commercial*	rec. centers*	groceries*
DMHI 2017-2013	0.19	0.20	0.19	0.24	0.23	0.18	0.19	0.22	0.23	0.20
train/bus stations	0.25	0.10	0.10	0.22	**0.27**	0.01	0.17	0.21	0.16	0.10
smartbus lines	0.24	0.00	-0.03	0.06	0.23	0.04	0.00	-0.04	0.20	-0.09
bike lanes	0.74	0.19	0.25	**0.43**	**0.34**	0.11	**0.33**	0.16	0.15	0.22
bus stops	**0.33**	-0.02	0.09	**0.58**	**0.50**	**0.31**	**0.35**	**0.55**	0.19	**0.40**
airports	0.07	0.03	0.05	0.08	0.01	-0.06	-0.13	-0.12	-0.16	0.08
toxic releases	0.17	**0.45**	**0.35**	0.22	-0.01	-0.11	0.05	0.11	0.02	0.15
railways	**0.35**	1	**0.67**	**0.44**	0.08	-0.07	0.14	0.26	**0.33**	**0.40**
AADT 5000	0.09	0.14	0.03	**0.35**	**0.42**	0.22	0.21	**0.35**	**0.27**	0.23
industry	**0.35**	**0.78**	0.62	**0.42**	-0.01	-0.04	0.10	**0.30**	0.25	**0.28**
brownfields	**0.41**	**0.45**	**0.39**	0.68	**0.35**	0.13	**0.43**	**0.50**	**0.32**	**0.44**
NAASO2	0.14	**0.40**	0.26	0.12	-0.09	0.01	0.09	0.00	**0.32**	0.20
poor pavement	**0.43**	0.08	0.17	**0.48**	1	0.19	**0.39**	**0.44**	0.25	0.19
demolitions	-0.09	-0.10	0.01	0.03	-0.05	-0.04	-0.04	-0.07	-0.21	0.13
vacancies	-0.07	-0.18	-0.12	0.09	0.03	0.02	-0.06	0.03	**-0.30**	0.22
public libraries	0.06	-0.07	0.01	0.24	0.19	1	**0.30**	0.25	0.20	0.18
schools	0.17	0.04	0.01	0.26	**0.37**	**0.34**	0.54	0.24	0.23	**0.33**
colleges	0.20	0.01	-0.04	0.21	0.25	-0.04	0.20	0.22	0.15	0.16
Head Start	0.01	0.10	0.13	0.12	-0.05	0.03	0.01	0.16	0.08	0.15
police stations	0.07	0.12	0.16	0.15	0.08	-0.01	0.04	0.13	0.06	0.11
fire stations	0.23	0.01	0.08	0.21	**0.29**	0.09	0.17	0.09	0.14	0.16
commercial	0.08	0.23	0.17	**0.36**	0.21	**0.29**	**0.28**	0.56	**0.32**	0.25
recreation centers	0.07	0.07	0.10	0.10	0.10	0.16	0.06	0.16	0.24	0.04
hospitals	0.03	-0.02	-0.12	0.05	0.12	0.06	0.13	0.07	0.10	0.04
health centers	0.19	0.08	0.07	0.16	0.15	**0.27**	**0.31**	0.18	0.17	0.14
groceries	0.08	0.00	0.06	0.24	0.07	**0.31**	**0.28**	0.18	0.10	0.25
parks	-0.09	-0.12	-0.12	-0.04	-0.15	0.02	0.02	-0.09	-0.05	-0.03
cemeteries	0.04	0.25	0.22	0.12	-0.04	-0.06	0.01	0.04	-0.01	0.15

*Quantified at the buffer level. Values in bold are correlations between distinct variables with p-values ≤ 0.001. Values in grey are correlations between scales of the same variable.

Table S3. Spearman correlation of select explanatory variables with all other covariates at the buffer scale.

covariate*	bike lanes*	railways	industry*	brownfields*	poor pavement	libraries	schools*	commercial*	rec. centers*	groceries*
train/bus stations	**0.47**	0.16	0.17	**0.43**	**0.41**	0.11	0.20	**0.32**	**0.33**	0.13
smartbus lines	**0.46**	0.04	0.07	0.19	0.30	0.03	0.13	0.07	**0.28**	-0.04
bike lanes	**1**	**0.35**	**0.46**	**0.64**	**0.43**	0.06	**0.38**	**0.31**	0.23	**0.43**
bus stops	**0.44**	0.02	0.22	**0.71**	**0.53**	0.20	**0.40**	**0.71**	0.18	**0.43**
airports	0.14	0.12	0.15	0.15	0.11	0.05	-0.06	-0.04	-0.23	0.19
toxic releases	**0.38**	**0.50**	**0.70**	**0.38**	0.15	-0.12	0.04	0.14	0.12	0.21
railways	**0.48**	**0.75**	**0.88**	**0.64**	0.14	0.04	0.20	**0.34**	**0.33**	**0.49**
AADT 5000	**0.28**	0.18	0.26	**0.68**	**0.47**	**0.27**	**0.44**	**0.71**	**0.31**	**0.45**
industry	**0.46**	**0.67**	1	**0.62**	0.17	0.01	0.18	**0.43**	**0.28**	**0.37**
brownfields	**0.64**	**0.44**	**0.62**	1	**0.48**	0.24	**0.54**	**0.74**	**0.41**	**0.62**
NAASO2	**0.27**	**0.40**	**0.28**	0.25	0.04	0.16	0.22	0.10	**0.40**	**0.28**
poor pavement	**0.59**	0.15	**0.30**	**0.73**	**0.75**	0.19	**0.50**	**0.62**	**0.29**	**0.35**
demolitions	-0.01	-0.10	-0.03	0.08	-0.05	0.05	0.06	0.05	**-0.28**	0.17
vacancies	-0.01	-0.13	-0.05	0.16	0.02	0.03	-0.04	0.13	**-0.32**	0.26
public libraries	0.15	0.04	0.02	**0.48**	**0.31**	**0.29**	**0.64**	**0.52**	0.30	**0.46**
schools	**0.38**	0.14	0.18	**0.54**	**0.39**	**0.30**	1	**0.44**	0.29	**0.49**
colleges	**0.36**	-0.11	-0.11	**0.44**	**0.43**	0.20	**0.47**	**0.43**	0.10	0.18
Head Start	0.01	0.26	0.24	0.14	-0.17	0.00	-0.05	0.20	0.10	0.26
police stations	0.18	**0.29**	**0.35**	**0.42**	0.10	0.08	0.00	**0.36**	0.00	0.22
fire stations	**0.58**	0.16	0.20	**0.50**	**0.46**	0.16	**0.41**	0.27	0.16	**0.32**
commercial	**0.31**	0.26	**0.43**	**0.74**	**0.44**	0.25	**0.44**	1	**0.48**	**0.43**
recreation centers	0.23	**0.33**	**0.28**	**0.41**	0.25	0.20	**0.29**	**0.48**	1	**0.41**
hospitals	0.13	-0.15	-0.20	0.20	**0.41**	0.20	**0.44**	0.16	0.18	0.06
health centers	**0.47**	0.17	0.09	**0.36**	**0.40**	0.14	**0.60**	**0.32**	**0.33**	**0.27**
groceries	**0.43**	**0.40**	**0.37**	**0.62**	0.19	0.18	**0.49**	**0.43**	**0.41**	1
parks	-0.20	-0.04	-0.06	-0.08	-0.12	0.05	0.22	0.00	0.09	-0.06
cemeteries	-0.01	0.20	**0.28**	0.12	-0.06	0.02	-0.05	0.08	0.04	0.07

*Quantified at the buffer level. Values in bold are correlations between distinct variables with p-values ≤ 0.001. Values in grey are correlations between scales of the same variable.

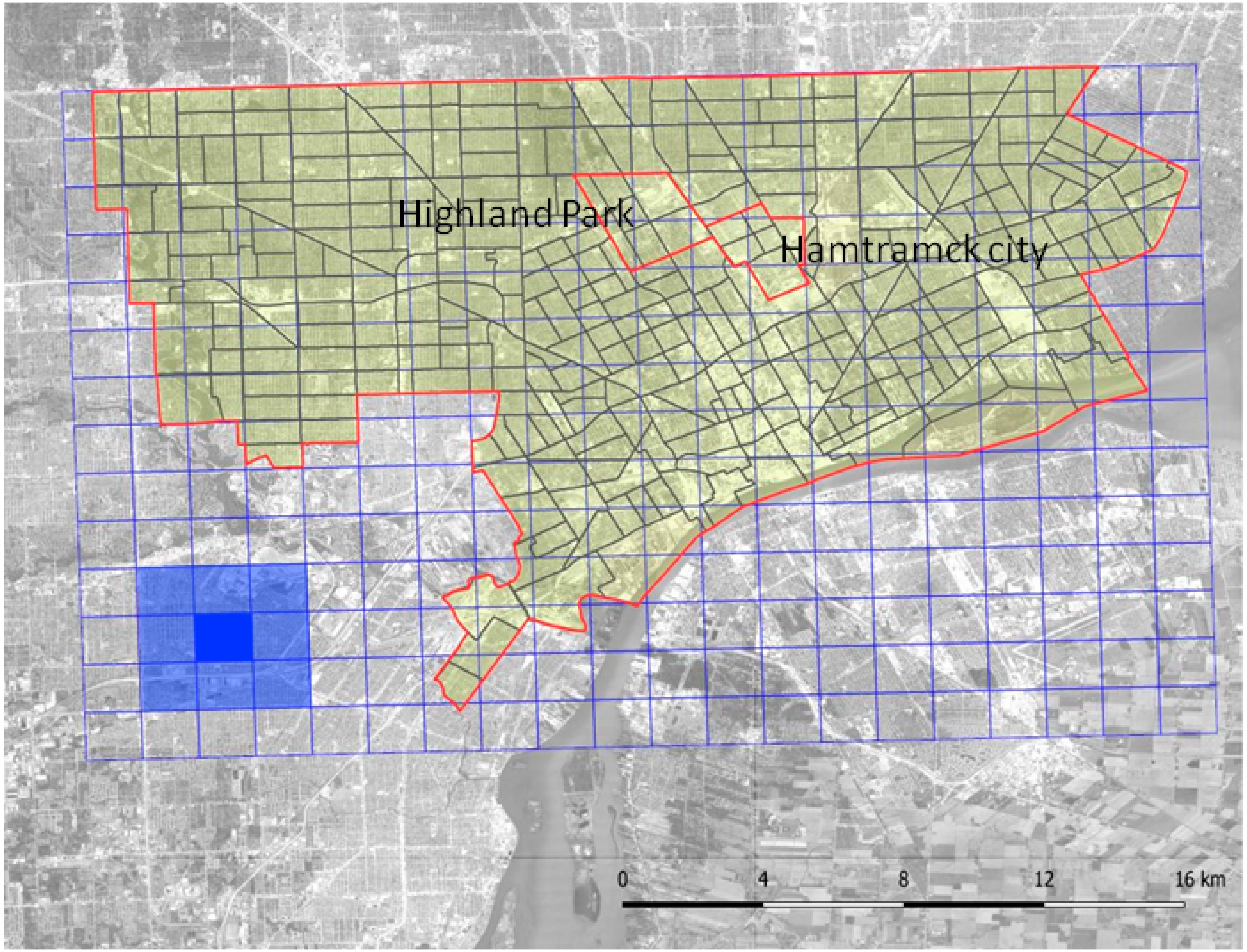

Figure S1. The study area of metropolitan Detroit, with census tract outlines overlaid by the 1x1 mile standardized grid of cells used for correlation analyses. The solid blue box shows the cell scale, while the shaded blue box shows the surrounding buffer scale. The red outline indicates the boundary of Detroit city, Highland Park city and Hamtramck city.

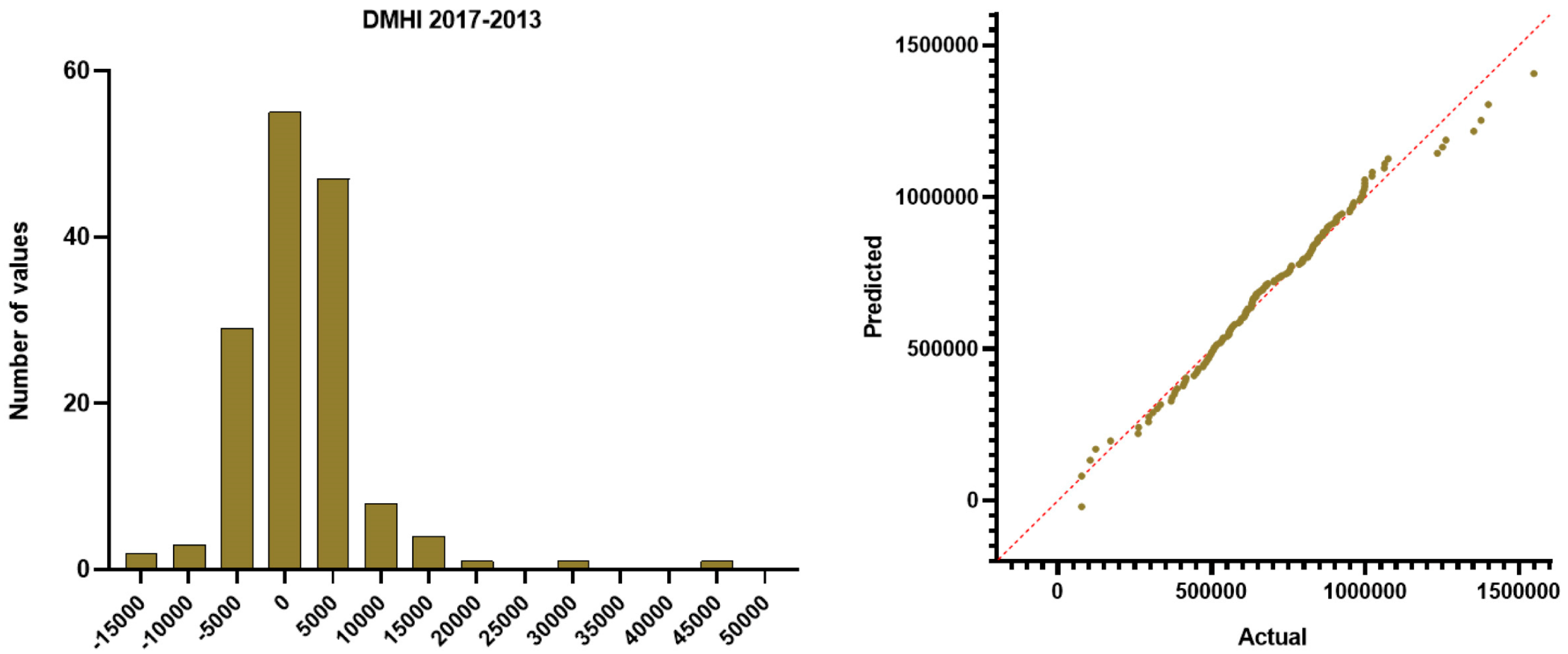

Figure S2. Histogram and normal quantile plot of the difference in median household income (DMHI) between 2017 and 2013.

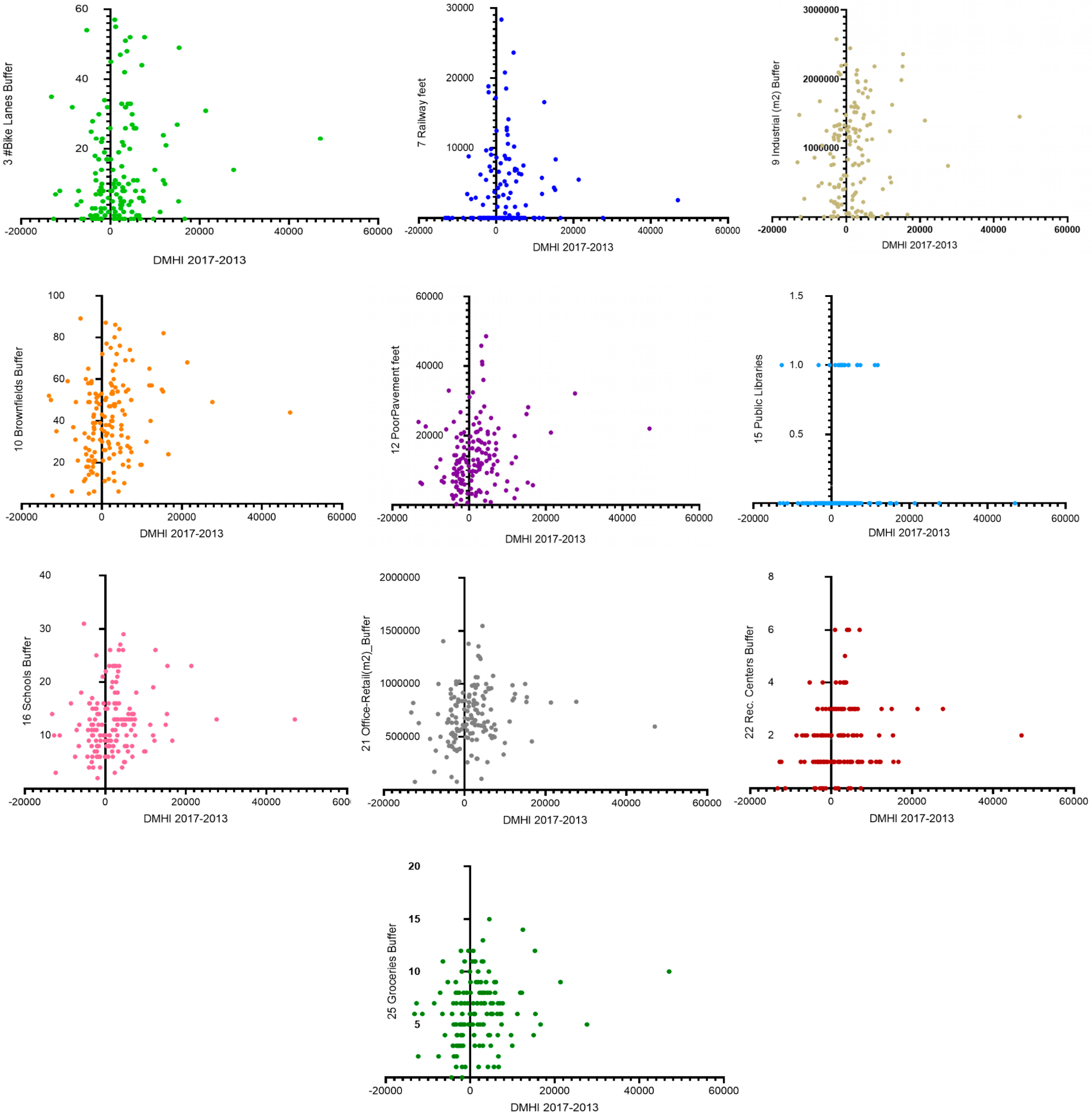

Figure S3. Scatter plots of DMHI and bike lanes, railways, industry, schools, brownfields, poor pavement, public libraries, office/commercial space, recreation centers and groceries.
